# Assessing the habitat suitability of agricultural landscapes for characteristic breeding bird guilds using landscape metrics

**DOI:** 10.1007/s10661-017-5837-2

**Published:** 2017-03-16

**Authors:** Friederike Borges, Michael Glemnitz, Alfred Schultz, Ulrich Stachow

**Affiliations:** 1 Leibniz Centre for Agricultural Landscape Research (ZALF), Eberswalder Str. 84, 15374 Müncheberg, Germany; 2 Eberswalde University for Sustainable Development, Alfred-Möller Str. 1, 16225 Eberswalde, Germany

**Keywords:** Landscape assessment, Bird monitoring, Binary logistic regression, Moving windows, Landscape composition, Landscape configuration

## Abstract

Many of the processes behind the decline of farmland birds can be related to modifications in landscape structure (composition and configuration), which can partly be expressed quantitatively with measurable or computable indices, i.e. landscape metrics. This paper aims to identify statistical relationships between the occurrence of birds and the landscape structure. We present a method that combines two comprehensive procedures: the “landscape-centred approach” and “guild classification”. Our study is based on more than 20,000 individual bird observations based on a 4-year bird monitoring approach in a typical agricultural area in the north-eastern German lowlands. Five characteristic bird guilds, each with three characteristic species, are defined for the typical habitat types of that area: farmland, grassland, hedgerow, forest and settlement. The suitability of each sample plot for each guild is indicated by the level of persistence (LOP) of occurrence of three respective species. Thus, the sample plots can be classified as “preferred” or “less preferred” depending on the lower and upper quartiles of the LOP values. The landscape structure is characterized by 16 different landscape metrics expressing various aspects of landscape composition and configuration. For each guild, the three landscape metrics with the strongest rank correlation with the LOP values and that are not mutually dependent were identified. For four of the bird guilds, the classification success was better than 80%, compared with only 66% for the grassland bird guild. A subset of six landscape metrics proved to be the most meaningful and sufficiently classified the sample areas with respect to bird guild suitability. In addition, derived logistic functions allowed the production of guild-specific habitat suitability maps for the whole landscape. The analytical results show that the proposed approach is appropriate to assess the habitat suitability of agricultural landscapes for characteristic bird guilds.

**Electronic supplementary material:**

The online version of this article (doi:10.1007/s10661-017-5837-2) contains supplementary material, which is available to authorized users.

## Introduction

The decline of biological diversity in European landscapes is well documented by many regional and Europe-wide studies (Defra 2013). Of all landscape types, agricultural landscapes are facing particularly pressing problems, as indicated by the strongest decline of indicator species, the most important being farmland birds (Defra 2013). This trend is projected to continue, as some studies show (Pereira et al. 2010; de Baan et al. 2013). A multitude of factors have been identified as contributing to farmland bird decline, especially factors related to the type and intensity of land use and management (Butler et al. 2010; Defra 2013; Langgemach and Ryslavy 2010). Some of these factors are difficult to measure, and many are correlated with each other. The problem of identifying the effects of the various driving forces is further complicated by the fact that the ongoing agricultural land use changes simultaneously act at multiple scales. They can be observed, for example, at the field scale (e.g. changes in the management of crop species) (Guerrero et al. 2012), at the farm scale (e.g. changes in crop rotations) (Smith et al. 2010) and at the landscape scale (e.g. simplified habitat diversity and geometries) (Danhardt et al. 2010). All these changes impact landscape composition (occurrence and frequency of different habitat types) and configuration (spatial arrangement of habitats). These effects can partly be expressed quantitatively with measurable or computable indices, i.e. landscape metrics. Landscape metrics can take into account structural landscape traits at different scales (Uuemaa et al. 2013), many of which are potentially important for farmland bird species. Landscape metrics also include landscape-wide information, thus covering the whole landscape. This is more advantageous than focusing on a selection of habitats that are considered relevant for the occurrence of specific bird species under study. A landscape-wide approach is supported by recent work conducted by Mimet et al. (2014). See also Fischer and Lindenmayer (2006), who refer to this as a “landscape-centred approach”, in contrast to the classical “habitat-centred approach”. Additionally, metrics allow generalization from the specific habitat types within specific study areas when habitat information for the metrics includes only the number, diversity, geometry and spatial configuration of the habitat types, but no ecological details pertaining to the specific habitat quality are available.

Suitable landscape data can be retrieved from digital maps, which are becoming abundant and available for many regions, based on aerial photos, satellite images, ground mapping, etc. Modern methods, including GIS and spatial statistics, allow for area-wide analyses of landscape metrics and, if time series are available, the characterization of trends. Because many of these data already exist, the calculation of landscape metrics is often well established (Uuemaa et al. 2013; Sundell-Turner and Rodewald 2008), demanding much less effort than the monitoring of farmland bird populations. In fact, while area-wide assessments of farmland bird occurrences, as indicators of biodiversity, are reasonable and desirable, equivalent bird monitoring schemes are out of reach because of prohibitively high resource needs. In this situation, it is important to derive and validate relationships between bird species occurrences and structural landscape features to consider landscape metrics as indicators of farmland bird occurrence.

We suggest that the combination of empirical bird occurrence data and landscape data (i.e. landscape metrics) has the potential to improve area-wide assessments of landscapes as habitat for birds. Thus, to contribute to the development of an appropriate methodology, we draw on an extensive set of bird occurrence data of an agricultural landscape (approximately 20,000 observations on the presence of species, with more than 42,000 observed bird individuals during 4 years of monitoring), and we test a variety of landscape metrics as potential indicators of defined groups of bird species.

Generalizations of case studies are needed to address the habitat suitability for birds on a landscape scale. What is the essence of locally detected relationships between bird occurrence and landscape characteristics that may be transferable to other regions? Answers to this question may help support the efficiency of empirical work (e.g. bird monitoring schemes), the design of meaningful biodiversity supporting schemes (e.g. changes in habitat configurations) and the comparison and assessment of landscape and land use development scenarios. While it is obvious that appropriate conservation means depend on the specific regional or local situation (Whittingham 2011), methods to generalize case study findings may be helpful to design local strategies. However, only a few attempts have been made to combine landscape metrics with generalized bird occurrence data (Hiron et al. 2015; Rüdisser et al. 2015).

Another important limitation of bird studies for more general assessments is exclusively focusing on selected species, as the analysis may be too focused on specific habitat needs, which are difficult to translate to other species. According to the niche concept, one single species can only serve as an indicator for a particular range of ecological conditions (Koskimies 1989). Habitats that seem unimportant for the selected species within the study area may be ignored, which also limits the ability to draw general conclusions for other landscape settings. A promising approach to generalize studies on bird occurrence at the landscape scale is the comprehensive analysis of well-defined species groups, composed of species with overlapping habitat requirements. These “bird guilds” automatically represent a more general type of relationship between birds and habitats. This approach is based on the possibility of identifying guilds as groups of species that require similar habitat requisites, depending on the research question (Brooks and Croonquist 1990; Flade 1994). Guilds can thus be classified according to various aspects, e.g. the representation of both specialists and generalists within one guild (O’Connell et al. 1998). The guild approach has been recommended by several authors for a more holistic assessment of bird communities (O'Connell et al. 2000; Karp et al. 2011; Marja et al. 2013), and guilds have been widely used as indicators of ecological conditions (O'Connell et al. 2000; Bryce et al. 2002), functional diversity (Tscharntke et al. 2008) and the biotic integrity of different land use systems (Karp et al. 2011).

Due to the year-to-year variability of spatial crop distribution patterns, the crop-related habitat configuration in agricultural landscapes is very dynamic. While the crop distribution patterns change from year to year, due to crop rotation, other landscape features are far more stable, e.g. most non-crop habitats. Hence, multiyear data of bird occurrences are needed to account for the spatial variability and to separate the effects of the yearly crop distribution from the effects of the more generic landscape configuration. It seems sensible to allow the variation of cropping patterns, i.e. the spatial effect of crop rotations, in the analysis of bird data because it reflects the very nature of agricultural landscapes, especially arable areas.

Examples of the potential of landscape metrics as indicators include conservation planning (Sundell-Turner and Rodewald 2008), habitat connectivity (Luque et al. 2012; Pascual-Hortal and Saura 2006), fragmentation (Fuentes-Montemayor et al. 2013), species richness in general (Schindler et al. 2013), gene flow (van Strien et al. 2014) and ecosystem services (Syrbe and Walz 2012; Frank et al. 2012). According to Uuemaa et al. (2013), the main focus of applying landscape metrics in previous years was the evaluation of changes in land use/land cover. The guild approach, in conjunction with landscape patterns, is regarded as useful to analyse the functionality, degree of degradation and restoration possibilities of different habitats in landscapes (van Halder et al. 2008; Bishop and Myers 2005; Melles et al. 2003).

Here, we present a methodological approach to relate bird occurrence data to landscape characteristics that may serve as an example to generalize and assess landscapes as habitats for birds. This approach combines the above-mentioned options to generalize case studies, i.e. defining guilds to represent the habitat requirements of biodiversity indicators; including all habitats within a landscape through the use of a digital “habitat map”, using only 12 habitat classes in our case; using metrics to express the landscape structure; and drawing on multiyear bird sample data. The combination of these components can represent a holistic, innovative methodological approach contributing to the current state of landscape suitability assessments for biodiversity indicators. The identification of relationships between bird occurrence and landscape structural characteristics may have the potential to:

1. Develop hypotheses that relate bird species occurrence probabilities to landscape metrics, to be tested in other landscapes
2. Allow assessments of landscapes in terms of bird habitat where no monitoring data are available
3. Explain the occurrence or absence of bird groups using landscape structural characteristics, and
4. Assess ex ante the effects of structural changes in agricultural landscapes on birds

Our specific objectives are to:

- Examine whether the classification of single bird species into bird guilds is a suitable approach for differentiating the habitat requirements of birds in landscapes
- Describe the statistical relationship between landscape structure, expressed using landscape metrics, and the suitability of agrarian landscapes as habitats for bird guilds
- Identify a particular set of landscape metrics for every specific guild that indicates the habitat suitability of the landscape

## Materials and methods

### Study area

**Fig. 1:**
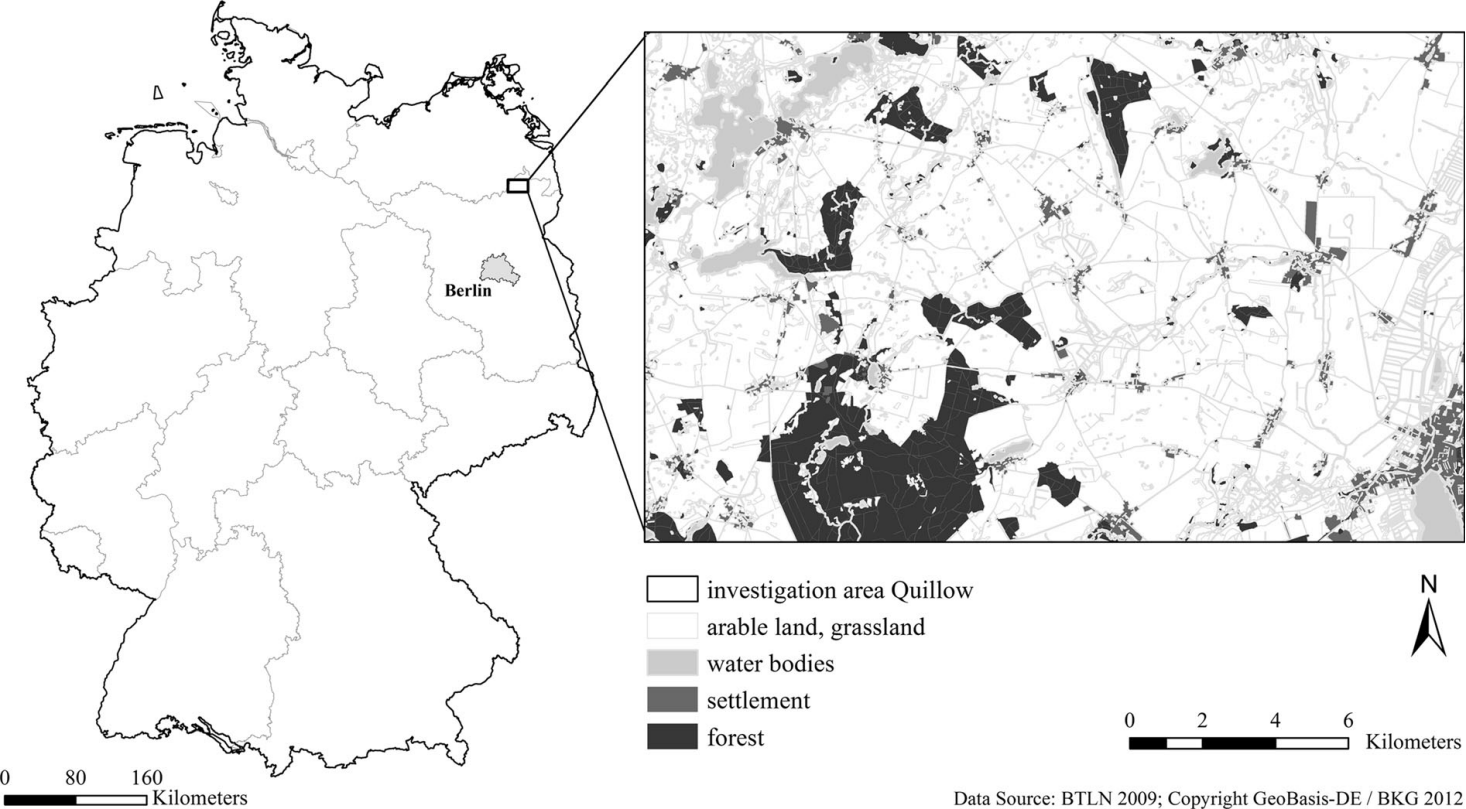
Location of the Quillow investigation area in the north-eastern German lowlands in the federal state of Brandenburg

The bird monitoring used in this study was performed in the AgroScapeLabs Quillow, a central observation area of the Leibniz Centre for Agricultural Landscape Research, which is located in the north-eastern German lowlands in the federal state of Brandenburg (Fig. 1). With a size of 290 km^2^, the investigation area includes the catchment of the Quillow, i.e. a small stream. The climatic conditions of the area are characterized by a transition between the mild oceanic and continental climate with hot and dry summers and cold winters. The long-term temperature mean measured from 1961 to 1990 varies between 7.6 °C in the west and 8.2 °C in the east of the area (Deutscher Wetter Dienst 2013). The average precipitation ranges between approximately 470 and 595 mm. The study area is shaped by the glaciations of the Pleistocene (Weichselian Glacial) and post-glacial processes. The soil conditions and landscape structure are typical for the northern portion of central continental Europe. The broad range in soil fertility and a highly heterogeneous distribution of different soil types determine the regional agricultural land-use capabilities. The elevation of the Quillow catchment is between 0 and 100 m above sea level, and the area is dominated by arable land (62.2%), accompanied by forests (12.7%), grasslands (11.9%) and other habitats. The area is typically “agricultural”, with soils of mostly medium and high yield potential (Stackebrandt and Manhenke 2002). Conventional farming is dominant, with an average farm size of 346 ha, and winter wheat and oilseed rape are dominant crops. The population density is quite low (36 inhabitants/km^2^), with a decreasing trend. Parts of the investigation area belong to a landscape conservation area. The significance of the region for birds is high, as BirdLife International identifies the region as one of the Important Bird and Biodiversity Areas (IBAs) in Europe (Heath et al. 2000).

### Bird observation data

**Fig. 2:**
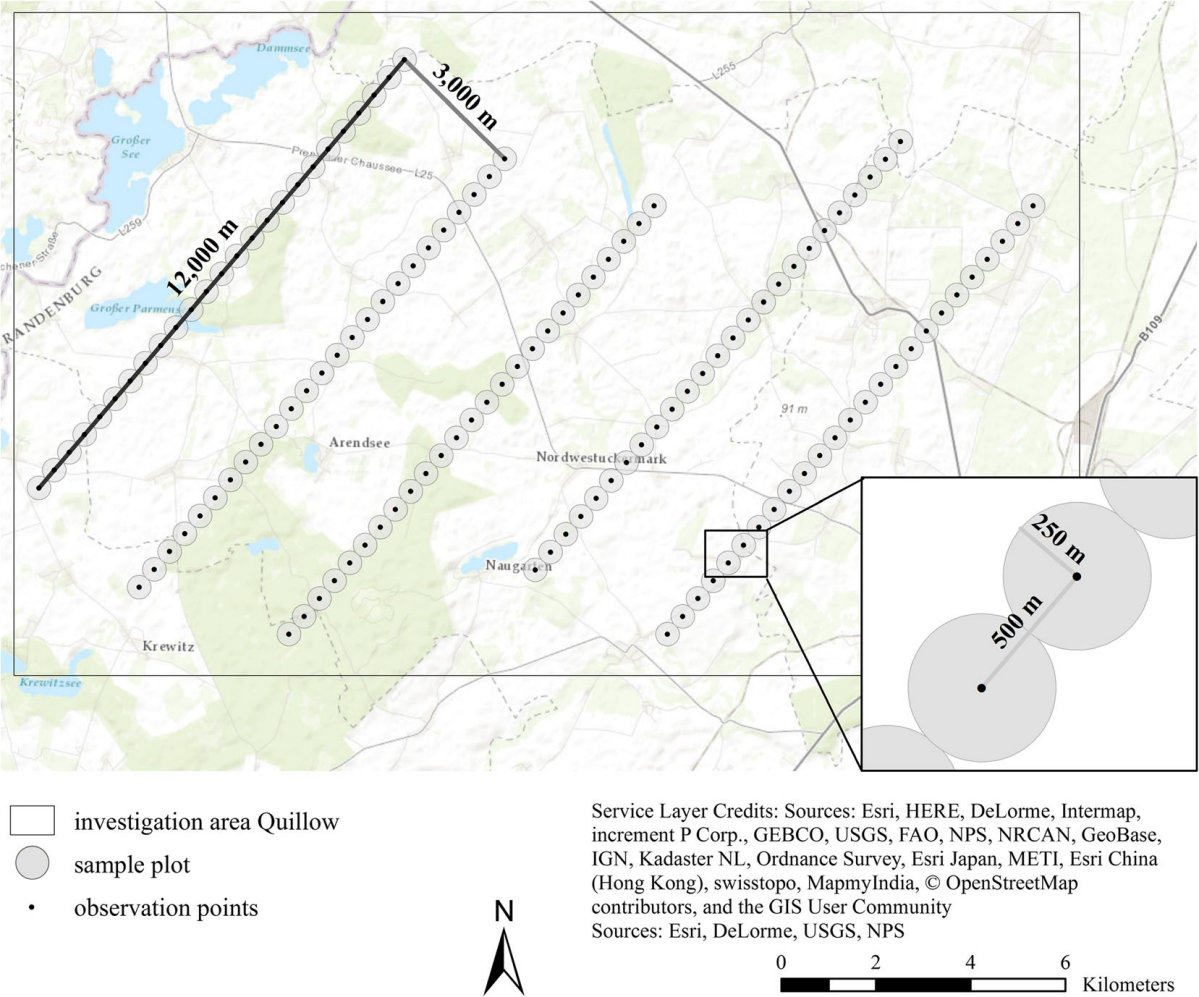
Design of the bird monitoring within the Quillow area

The bird monitoring was performed from 1999 to 2002 using the point-count method (Drapeau et al. 1999) (Fig. 2). Bird surveys were applied five times per year within the main breeding period, from April to June, with intervals of approximately 2 weeks. In 1999, only four observations took place. It is assumed that these numbers of surveys per year cover the actual occurrence of breeding birds in this study area. A regular sample grid of 125 observation points was chosen. On each of five straight parallel transects with a distance of 3000 m, 25 sample points with a distance of 500 m on the transect were selected. The same points were used every year. The basic principle of this method is the stationary recording of species by direct sight and/or sound for 10 min at the sample point. The observations were assigned to three experienced ornithologists and began in the early morning (5 a.m. to 8 a.m., depending on the season) and were finished not later than at 1 p.m. The observation points at one line were visited in a systematic way one after another. However, to avoid rough systematic errors, the surveys during the season started from different ends of the transects, and the transects were judiciously changed among the ornithologists within the years. Although the sampling approach was not completely random, the collected observation data may be considered representative for the study area because of the density and allocation of observation points and the frequency of observations. To avoid double counts from adjacent observation points, only those individuals were documented that occurred within a maximum distance of 250 m. In the following, the term “sample plot” is used for a circular area of ca. 20 ha with the observation point in the centre and a radius of 250 m. Every point represents an independent sample plot and is individually documented by an acquisition sheet as well as its location in coordinates to ensure reproducibility and comparability. From the multitude of data collected at each count, we use here only three: bird species, occurrence (presence/absence) and date.

### Data analysis

**Fig. 3:**
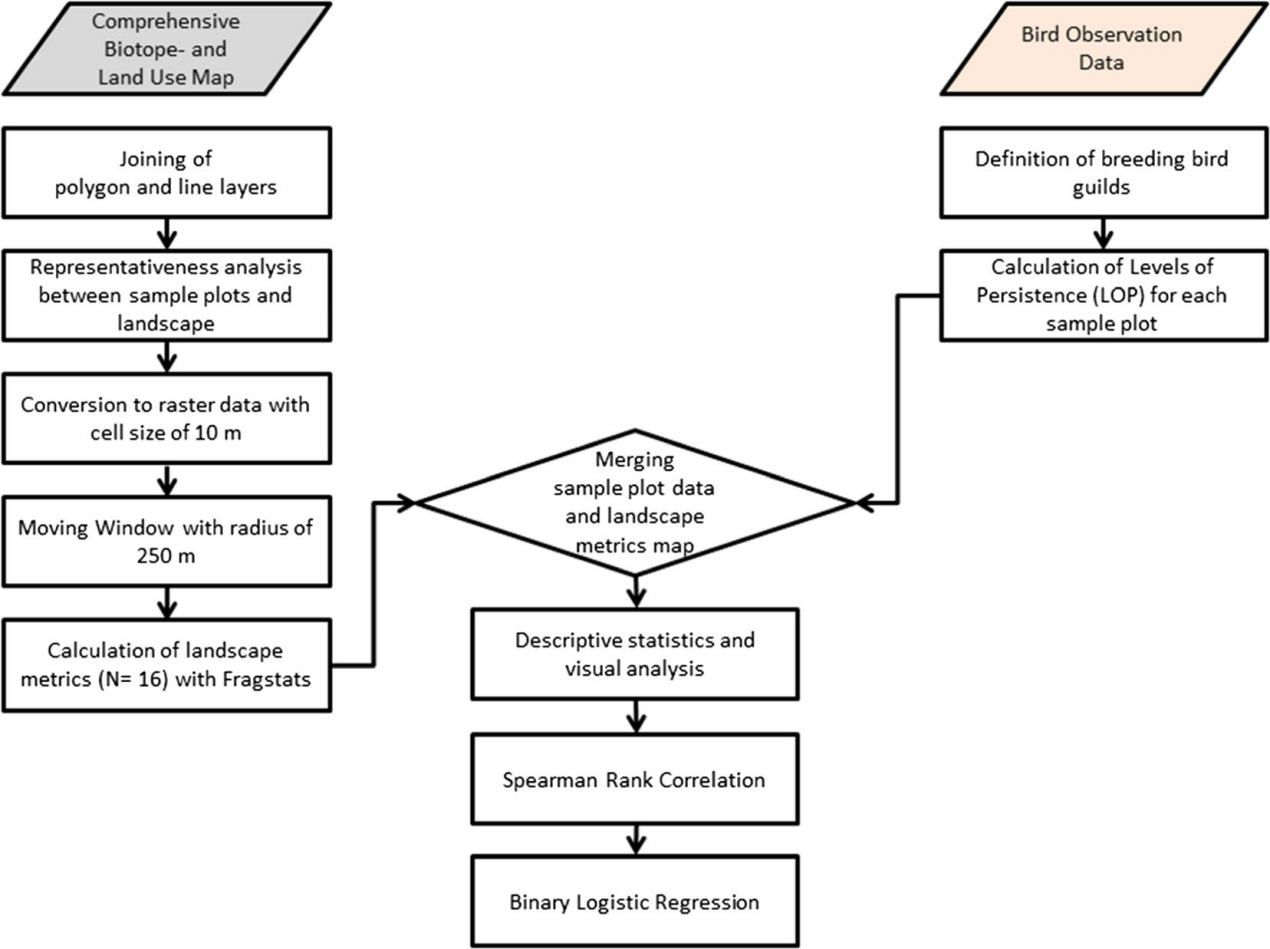
Analytical framework for analysing the relationship between bird occurrence and landscape structure

The procedure for the data analysis is summarized in Fig. 3. Detailed information is presented in the subsequent chapters as well as in the Online Resource (Fig. A 1).

### Definition of breeding bird guilds

The definition of breeding bird guilds with different habitat requirements focuses on the typical components that constitute rural landscapes in the area, with the aim to cover most of the typical habitat types and common bird species identified in the bird survey. The guild classification corresponded to the following five main habitat types typical of agricultural landscapes: farmland, grassland, hedgerow, forest and settlement. The species selection for a particular breeding bird guild refers to species selected in the National Strategy on Biological Diversity of Germany (BMU 2007) and to classifications in the literature (Table 1), as far as possible. Three typical species were chosen to characterize a specific guild. The selection additionally depended on the species’ frequency in the bird monitoring data. Only frequent and persistent species were selected. In Table 1, the guilds and three respective bird species are listed. For each guild, maps describing the spatial distribution of their occurrence, their abundance and the level of persistence (LOP) of occurrence values are shown in the Online Resource (Fig. A 2–A 6).

**Table 1:** Assignment of three bird species to guilds in the study area

Guild	Representative species		Reference	Presence^a^ in the Quillow observation area
	English name	Scientific name		
Farmland	Eurasian skylark	*Alauda arvensis*	Lutze et al. (2010); Gödeke et al. (2010)	100%
	Yellow wagtail	*Motacilla flava*	Lutze et al. (2010)	79%
	Corn bunting	*Emberiza calandra*	Lutze et al. (2010); Gödeke et al. (2010)	100%
Grassland	Meadow pipit	*Anthus pratensis*	Lutze et al. (2010); Hoffmann (2011)	84%
	Whinchat	*Saxicola rubetra*	Gödeke et al. (2010); Hoffmann (2011)	74%
	Northern lapwing	*Vanellus vanellus*	Flade (1994); Hoffmann (2011)	100%
Hedgerow	Red-backed shrike	*Lanius collurio*	Flade (1994); Lutze et al. (2010)	58%
	Yellow hammer	*Emberiza citrinella*	Flade (1994); Lutze et al. (2010)	100%
	Eurasian chaffinch	*Fringilla coelebs*	Flade (1994); Lutze et al. (2010)	100%
Forest	Wood nuthatch	*Sitta europaea*	Flade (1994); Gödeke et al. (2010)	100%
	Coal tit	*Parus ater*	Flade (1994); Gödeke et al. (2010)	100%
	Marsh tit	*Parus palustris*	Flade (1994); Gödeke et al. (2010)	79%
Settlement	Blue tit	*Parus caeruleus*	Lutze et al. (2010)	100%
	Great tit	*Parus major*	Lutze et al. (2010)	100%
	Eurasian tree sparrow	*Passer montanus*	Flade (1994)	100%

^a^Relative occurrence out of 19 separate investigation dates (19 = 100%)

### Description of bird occurrences

The occurrence of a guild at a sample plot is expressed by the presence (1) or absence (0) of the three characteristic species, neglecting the actual number of individuals. The occurrences were summarized as follows: in each of the surveys, a maximum of three was possible on each sample plot when all species of the guild could be observed, and 0 when no species was observed. These values are taken to construct a so-called level of persistence (LOP) for every guild at each sample plot, which is the sum of all presence/absence values over the whole survey area and duration, with a theoretical minimum of 0 and a theoretical maximum of 57 (3 years with five surveys, 1 year with four surveys and three species per guild). It is true that the LOP approach introduced here has no direct ecological meaning. Due to its construction, it may be considered a further development of the common presence-absence approach. The addition of the frequency of bird occurrences at particular points may furthermore indicate the habitat quality of the sample areas in an aggregated quantitative way. A high LOP value means that species of a guild are frequently observed, so the area is considered to be a rather preferred habitat of that guild. The LOPs were derived for each of the five guilds, and statistical analysis was conducted for each guild for all sample plots. In total, 120 of the 125 potential sample plots were visited at all occasions and used for the analysis. Five sample plots were excluded due to missing data.

### Spatial landscape data

Data analyses were conducted using SPSS Statistics 22 software from IBM and ArcMap 10.2.2 software from ESRI. Landscape metrics were calculated based on the spatial data of the “Comprehensive Biotope and Land use Map” of the Federal State of Brandenburg (MLUL 2014). These classified data are based on colour infrared aerial photographs from 2009 combined and harmonized with data from 1995 (Schade et al. 2014). The biotope map from 2009 was chosen because of relevant improvements in the consideration of linear biotopes compared to the map from 1995. Because no significant changes in main land use structures between the two biotope mapping reference years occurred, it is assumed that the improved biotope map from 2009 represents the structural conditions in the landscape during the bird data collection period (1999–2002). The utilized interpretation key consists of 12 main land cover classes for the Federal State of Brandenburg (Walter 2012), of which 11 occur in the investigation area (Table 2) and are represented on the map as polygons or lines (flowing waters, standing water bodies, hedgerows and tree rows, built-up areas and traffic facilities).

**Table 2:** Biotope types covered by the sample areas (sample plots with a 250-m radius) in the study location (Quillow catchment)

Land cover classes	Land cover distribution Quillow sample plots
Flowing waters	0.00%
Standing waters	0.86%
Ruderal vegetation	1.49%
Bogs and marshes	0.78%
*Grasslands*	*6.88%*
Bushes, tree rows	1.04%
*Forests*	*17.59%*
*Arable land*	*69.62%*
Urban green and open space	0.5%
Special biotopes	0.00%
Built-up areas, traffic facilities	1.19%

The italic entries are the 3 biggest landcover classes

Habitat data were pre-processed, i.e. the polygon and line layer were joined to cover the area as accurately and in as much detail as possible. For this purpose, line data were buffered with a buffer distance of 5 m to ensure that line elements were not discarded during the conversion from polygon to raster format. The processed spatial information had a standard deviation of 0.99% with respect to the original polygon data. The standard deviation of the habitat coverage and original and combined data sample plots was less than 1% and was 0.92% smaller than the whole sample area. The sample plots were tested for representativeness using the biotope inventory of the whole area. The total classification comparison revealed a significant correlation of 0.993 between the sample plots and the total Quillow investigation area (original and combined data set), which was confirmed by Spearman’s rank correlation coefficient at the 5% error level. Therefore, the sample plot area was considered to represent the entire Quillow observation area reasonably well (Table 2).

### Landscape metrics

The application of landscape metrics was realized by using the raster version of the environmental software package FRAGSTATS, version 4.2 (McGarigal 2015). For this purpose, all vector data were converted to raster data with a target cell size of 10 m, and all calculations were accomplished using raster data with this cell size. The cell size of 10 m was considered appropriate to display the most suitable habitats (e.g. hedgerows) as very important, whereas small habitat fragments of only a few square metres were neglected. While converting from vector to raster data, the dominant biotope type was assigned to the target raster cell. The spatial data processing during the whole analysis is illustrated in the Online Resource to this paper (Fig. A 1).

To establish a spatial relationship between the landscape metrics and the occurrence of breeding bird guilds in individual sample plots, the landscape metric values were calculated using the exhaustive sampling moving window strategy. The moving window strategy is a well-known geographical method for creating continuous crossovers between distinct map cells (Hoffmann et al. 2016). A circle with a radius of 250 m for the moving window was selected due to the 500 m distance between neighbouring observation points. Consequently, a unique environment could be assigned to any sample plot. The result of applying this moving window procedure is a continuous surface for each metric, which reflects how an organism with that perceptual ability would perceive the structure of the landscape as measured by that metric (McGarigal 2015). The border of the investigation area was not treated as an edge or border during the analysis. The eight-cell neighbourhood rule was used to identify connected raster cells of the same type (e.g. all surrounding cells were considered).

In this study, an initial selection of 16 different landscape metrics was performed according to their utility for landscape assessment, their coverage of different structural landscape aspects and their potential relevance for wildlife resources (Trani and Giles 1999). The initial selection covered the main properties of habitat composition and configuration. The following indices were considered: connectance index (CONNECT), effective mesh size (MESH), interspersion juxtaposition index (IJI), landscape division index (DIVISION), landscape shape index (LSI), number of patches (NP), patch cohesion index (COHESION), patch density (PD), percentage of like adjacencies (PLADJ), edge density (ED), largest patch index (LPI), patch richness density (PRD), Simpson’s diversity index (SIDI), contiguity index distribution (CONTIG_MN), perimeter-area fractal dimension (PAFRAC) and shape index distribution (SHAPE_MN). To calculate values for each sample plot, the metric values were averaged within the sample plot boundary (Fig. A 1 in the Online Resource). Table 3 explains the interpretation of the six indices that were entered into the final statistical models for the different bird guilds. The FRAGSTATS manual (McGarigal 2015) contains additional information on all 16 metrics.

**Table 3:** Meaning and interpretation of the landscape metrics (abbreviations in brackets) included in the final model solutions (McGarigal 2015)

Metric	Unit	Description
Contiguity index distribution (CONTIG_MN)	None 0 ≤ CONTIG_MN ≤ 1	Assesses the spatial connectedness, or contiguity, of cells within a patch to provide an index on patch boundary configuration and thus patch shape (LaGro 1991); CONTIG equals 0 for a one-pixel patch and increases to a limit of 1.
Edge density (ED)	m/ha	ED = 0 when there is no edge in the landscape, that is, when the entire landscape and landscape border, if present, consists of a single patch.
Largest patch index (LPI)	%	The largest patch index quantifies the percentage of total landscape area comprised by the largest patch. As such, it is a simple measure of dominance.
Patch density (PD)	*N*/100 ha	Patch density has the same basic utility as number of patches as an index, except that it expresses number of patches on a per unit area basis that facilitates comparisons among landscapes of varying size.
Shape index distribution (SHAPE_MN)	None SHAPE_MN ≥ 1	SHAPE = 1 when the patch is square and increases without limit as the patch shape becomes more irregular. On the landscape level, the mean of all patches in the landscape is calculated.
Simpson’s diversity index (SIDI)	None 0 ≤ SIDI ≤ 1	SIDI = 0 when the landscape consists of only 1 patch (i.e. no diversity). SIDI approaches 1 as the number of different patch types increases and the proportion of the area among patch types becomes more equitable.

### Descriptive statistics and visual analysis

To obtain a visual overview of the bird sample data and to describe the validity of the data, various descriptive statistics were performed. Bird population abundances are influenced by several external factors, such as habitat quality, resource availability and species-related reproductive differences, which are not included in our data. The data from particular sampling events were summarized to obtain information on how many bird species were present in the whole observation area per year and to compare the years. Hence, relatively permanent and temporarily occurring bird species could be identified, as could trend information over individual years (Table 4). The breeding bird monitoring resulted in a total of 135 bird species sighted over the 4 years of observation, with a total number of 42,361 bird individuals observed during 20,007 sightings.

**Table 4:** Annual breeding bird monitoring values

Year	Number of species	Species cumulative values	Sum of bird sightings
1999	108	108	4,043
2000	114	124	5,315
2001	115	132	4,961
2002	112	135	5,688
Total			20,007

**Fig. 4:**
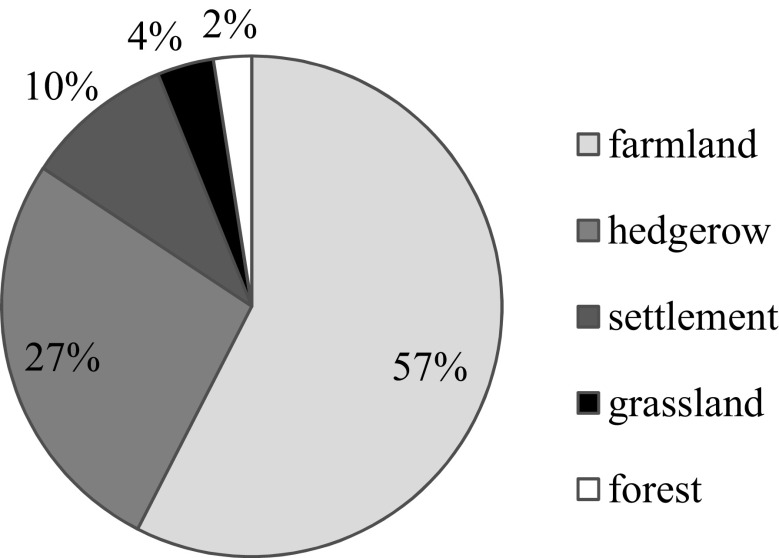
Percentage distribution of breeding bird guilds related to representative species presence

The breeding bird guild representatives selected for our analyses sum to 17,549 individual observations (41% of the total) registered during 8,432 listed individual sightings; the 15 selected species represent 42% of the observed individuals of the overall bird community. The composition of the different guilds is shown in Fig. 4. Farmland birds constituted the largest group, with a share of 57%, followed by the hedgerow species, with 27%. The numbers underpin the prior characterization of the investigation area as an agricultural landscape. The detailed information for the particular bird species used in our analysis as guild representatives is shown in Table 1. The individual plot observation results were then further processed per guild to obtain information on the LOP for each sample plot.

**Fig. 5:**
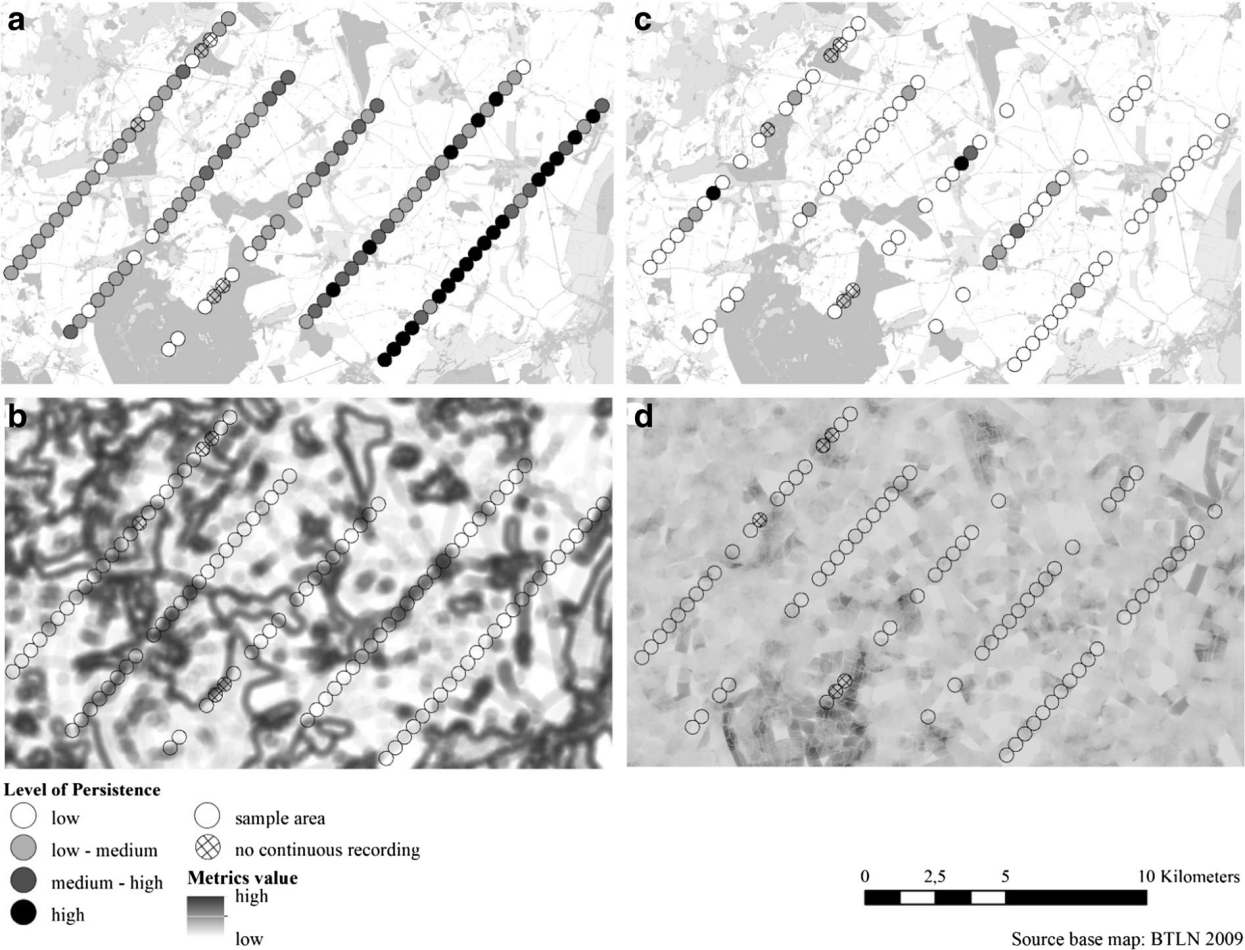
Distribution maps for the farmland (**a**, **b**) and grassland (**c**, **d**) guild occurrence in the investigation area in combination with landscape metric maps: **a** level of persistence (LOP) for the farmland guild; **b** farmland bird occurrence plotted against Simpson’s diversity index landscape metric; **c** LOP for the grassland guild; **d** grassland bird occurrence plotted against the shape mean landscape metric

To obtain a visual impression of the spatial distribution of the selected bird guilds in the investigation area, the occurrence information for the guilds in the sample plots was displayed on spatial maps of related landscape metrics. Figure 5 contrasts the spatial distribution of the farmland and the grassland guild together with the distribution of the landscape metric with the statistically strongest relationship. A version of Fig. 5 coloured for better visual differentiation is available in the Online Resource (Fig. A 7).

### Analytical statistics

The main objective of the statistical analysis of the bird occurrence data was to determine whether it was possible to predict the guild occurrence within the sample areas based on the values of the specific landscape metrics around the sample areas. The occurrence of a guild in a sample plot was expressed by the occurrence of its 3 characteristic species and their LOP values. The derived LOP values serve as a target variable for subsequent analytical statistics. Due to the statistical properties of the LOP values and of the considered landscape metrics, only analytical methods without special requirements concerning the statistical distributions of the included features were applied.

The pairwise relationships between landscape metrics and the LOPs for the particular guilds were analysed with Spearman rank correlation coefficients. The classification of “preferred” and “less preferred” habitats was then accomplished with a binary logistic regression using input data derived from the LOPs. The binary logistic regression was selected as a principal analytical method because the analytical focus was on the qualitative distinction between rather unsuitable habitats (less preferred) and rather suitable habitats (preferred) based on landscape metrics. The binary logistic regression model is able to predict a binary response dependent on one or more independent inputs and has no particular requirements concerning the scale level of these inputs. Practically any appropriate quantitative or qualitative variable including landscape metrics may serve as input. The binary logistics regression allows a direct and transparent interpretation of input effects, and it is furthermore possible to evaluate the reliability of the classification and to rank the importance of inputs. Mathematically, the binary logistic regression function takes values between 0 and 1, and the binary response is assigned to the function values using a cut point between 0 and 1. In most practical situations, 0.5 serves as the cut point. Mathematical details and the implementation in SPSS are described by Field (2013).

Here, the binary response (preferred vs. less preferred habitats) is derived from the LOPs as a dependent variable, and a set of landscape metrics is used as independent variables. Among the landscape metrics, those three metrics were selected as inputs that showed the greatest Spearman correlation coefficient with the particular LOP. In case of obviously functionally dependent landscape metrics (absolute values of pairwise correlation coefficients equal to or close to 1), only one of them was used as input in order to keep the inputs as mutually independent as possible. The number of input variables was limited to three to enable a transparent interpretation of the mutual interconnections.

The members of the two alternative classes (preferred vs. less preferred habitats) were taken from the cases that fell below the 25% quartile of the LOPs and above the 75% quartile of the LOPs of every particular guild and observation point. The “lower” class indicates rather unsuitable habitats (less preferred), and the “upper” class indicates rather suitable habitats (preferred). Each class contains approximately 30 cases. Within SPSS, the binary logistic regression was executed with the variable selection method “Enter”, i.e. all independent input variables are entered in a single step (Field 2013). To evaluate and compare the general classification success and the goodness of fit of the logistical regression models, the share of correct classifications was determined and Nagelkerke *R* ^2^ values were calculated.

The statistical significance of individual input variables in the guild models is derived from the *p* value of the Wald chi-square statistics. The *p* values indicate the strength of evidence of the relationship between target and input (Thompson 2009). To interpret the effect of inputs (the various landscape metrics) as having the same scale, standardized regression coefficients were calculated according to the method proposed by King (2007). The absolute values of standardized coefficients indicate stronger predictors in the equation.

## Results

### Correlation analysis

The relationships between bird occurrence and landscape structure were first analysed in a univariate way, testing the correlation between the 16 particular landscape metrics and the LOP values for the 5 different breeding bird guilds. Table 5 summarizes the results of the correlation analysis for the five guilds. The table contains the three landscape metrics that showed the strongest correlation with the particular target variable LOP.

**Table 5:** Spearman rank correlation coefficients

Target variable	Most correlated landscape metrics
LOP of farmland guild	SIDI (−0.388)** SHAPE_MN (−0.352)** ED (−0.288)**
LOP of grassland guild	SHAPE_MN (−0.187)* CONTIG_MN (−0.172) PD (0.144)
LOP of hedgerow guild	SIDI (0.625)** LPI (−0.620)** ED (0.580)**
LOP of forest guild	SHAPE_MN (0.534)** SIDI (0.413)** ED (0.336)**
LOP of settlement guild	SIDI (0.703)** ED (0.664)** LPI (−0.614)**

Spearman correlation coefficient values are presented in parentheses; the statistical significance is also indicated; for landscape metric abbreviations, see Table 3

*Statistically significant at the 5% error level; **Statistically significant at the 1% error level

For example, the three most strongly correlated landscape metrics of the farmland guild are Simpson’s diversity index (SIDI), the shape index distribution (SHAPE_MN) and the edge density (ED). All three act in the opposite direction of the LOP and are statistically significant at the 1% error level, that is, the higher the value of the metrics, the lower the occurrence of farmland birds. Greater *diversity*, greater *shape mean* and greater *edge density* were correlated with the reduced occurrence of typical farmland birds.

The correlation analysis results of the other four guilds can be interpreted in an analogous manner. With the exception of the grassland bird guild, all selected landscape metrics were significantly correlated with the particular guild LOP. In the case of the grassland birds, the only significant landscape metric was the *shape mean*, with a 5% error.

### Binary logistic regression

Table 6 summarizes the results of the executed logistic regressions with class values derived from the particular LOP values as the target variable and the three most strongly correlated landscape metrics as independent variables. In addition to the rate of correct classifications, the Nagelkerke *R* ^2^ value is reported. Additionally, the output of the logistic regression indicates what type of misclassification occurs, i.e. whether there is a systematic preference among misclassifications for preferred or less preferred areas.

**Table 6:** Overview of classification results

Guild	Correct classifications in %	Nagelkerke *R* ^2^	Remark
Farmland	85.70	0.588	Less preferred areas are overestimated
Grassland	66.20	0.225	Less preferred areas are overestimated
Hedgerow	83.30	0.535	Less and more preferred areas are misclassified in equal shares
Forest	91.70	0.883	More preferred areas are slightly overestimated
Settlement	89.60	0.739	Less and more preferred areas are misclassified in equal shares

For four of the five considered guilds, it was possible to distinguish between less preferred and preferred habitats with success rates better than 80%. Only in the case of the grassland guild was the rate of correct classifications (66%) apparently poorer. Because an identical number of cases was used for all five guilds, it was possible to use the Nagelkerke *R* ^2^ value in a comparative test of the classifications. It was not possible to derive the overall statistical significance of the classification, but it was possible to use the Nagelkerke *R* ^2^ value to interpret the reliability of the classification. In this respect, it was obviously easier to classify the preferred and less preferred habitats for species of the forest and settlement guilds than for species of the grassland and hedgerow guilds. The rate of correct classifications and the considerably lower Nagelkerke *R* ^2^ value for the grassland guild indicate that the classification of grassland habitats seems to be the most difficult.

Table 7 summarizes the detailed results for the farmland guild. In addition to the used input variables and the estimated unstandardized regression coefficients, the table shows the standardized regression coefficients and the *p* value of the Wald statistic. The latter two provide information concerning the relative importance of individual input variables (i.e. habitat suitability expressed by landscape metrics) and their statistical significance. The input variable acts independently of the input variables. In the estimated logistic regression function for the farmland guild, all three unstandardized regression coefficients were statistically significant at the 5% error level. The standardized regression coefficient for Simpson’s diversity index (SIDI) indicates that this landscape metric had the greatest relative importance. In the case of identical relative changes among the three inputs, changes in the SIDI would have the greatest effect.

**Table 7:** Result of habitat classification of farmland guild using binary logistic regression

Farmland guild			
Input variable	Unstandardized regression coefficient	Standardized regression coefficient	*p* value of Wald chi-square statistics
SIDI	−15.733	−0.649	0.001
SHAPE_MN	−2.446	−0.336	0.001
ED	0.027	0.448	0.018
Constant	5.338	–	0.001

Tables 8, 9, 10 and 11 summarize the details of the classification of the other considered guilds. All four tables follow the same structure, and the presented numbers may be interpreted in an analogous manner. The statistical significance of individual input metrics can be inferred from the *p* value of the Wald chi-square statistic (if <0.05, then statistical significance occurs at the 5% error level). The relative importance of the input metrics can be derived from the standardized regression coefficient (the greater the absolute value, the greater the relative importance).

**Table 8:** Result of habitat classification of grassland guild using binary logistic regression

Grassland guild			
Input variable	Unstandardized regression coefficient	Standardized regression coefficient	*p* value of Wald chi-square statistics
CONTIG_MN	−6.988	−0.680	0.019
SHAPE_MN	−1.637	−0.636	0.030
PD	−0.015	−0.210	0.150
Constant	6.936	–	0.100

**Table 9:** Result of habitat classification of hedgerow guild using binary logistic regression

Hedgerow guild			
Input variable	Unstandardized regression coefficient	Standardized regression coefficient	*p* value of Wald chi-square statistics
SIDI	4.992	0.257	0.345
LPI	−0.046	−0.234	0.434
ED	−0.001	−0.020	0.913
Constant	2.313	–	0.702

**Table 10:** Result of habitat classification of forest guild using binary logistic regression

Forest guild			
Input variable	Unstandardized regression coefficient	Standardized regression coefficient	*p* value of Wald chi-square statistics
SHAPE_MN	11.605	0.938	0.011
SIDI	35.493	0.920	0.008
ED	−0.053	−0.674	0.021
Constant	−25.263	–	0.012

**Table 11:** Result of habitat classification of settlement guild using binary logistic regression

Settlement guild			
Input variable	Unstandardized regression coefficient	Standardized regression coefficient	*p* value of Wald chi-square statistics
SIDI	15.650	0.653	0.008
LPI	0.073	0.332	0.225
ED	0.017	0.299	0.239
Constant	−12.289	–	0.058

Of the 16 different landscape metrics initially chosen, only 6 were retained in the final statistical models describing the preferred vs. less preferred habitats for the five selected bird guilds. The habitats for farmland and forest guilds as well as for hedgerow and settlement guilds were described with the same set of indices but with varying directions and combinations. Table 12 summarizes the selected landscape metrics together with the direction of their impacts.

**Table 12:** Landscape metrics and their relationships with the occurrence of five bird guilds

Metric	Guild				
	Farmland	Grassland	Hedgerow	Forest	Settlement
SIDI	↓		↑	↑	↑
SHAPE_MN	↓	↓		↑	
ED	↑		↓	↓	↑
CONTIG_MN		↓			
PD		↓			
LPI			↓		↑

The direction of the arrows indicates the direction of the correlation; a parallel increasing (↑) or contrary increasing (↓) relationship

## Discussion

The relationship between landscape structure and biota has been extensively described mostly for species richness issues, including birds (Atauri and de Lucio 2001; Morelli et al. 2013). With respect to birds, numerous studies emphasize the importance of landscape complexity (Fischer et al. 2011; Rüdisser et al. 2015) or heterogeneity (Morelli et al. 2013) for high species richness values. However, the “the more, the better” conclusion for the relationship between landscape heterogeneity and bird species richness in many of these studies is ultimately not readily applicable for conservation measures, for multiple reasons. One reason is the quantification problem for the target of conservation measures (“How much heterogeneity is enough?”); another is the disregard of the natural landscape setting (“What is the benchmark?”). Landscapes that differ in abiotic characteristics and land use histories also vary with respect to their species inventories and their ecological potentials and are therefore difficult to compare.

Some recent studies have made it clear that addressing species diversity or habitat suitability requires different sets of landscape metrics. Schindler et al. (2013) found that metrics quantifying patch shape, proximity, texture and landscape diversity are most suitable for describing species richness (species diversity), while those describing patch area, similarity and edge contrast rarely contribute to significant models for species diversity. In their review of the use of landscape spatial metrics as indicators for various ecosystem functions, Uuemaa et al. (2013) found that metrics describing landscape diversity, complexity or even fragmentation are the most relevant for effects on species and genetic diversity for different organism groups. Many studies stress that the abundance of particular bird species or species groups responds more strongly to the composition of land cover classes or even specific habitats than to metrics addressing landscape configuration (Uuemaa et al. 2013; Carrara et al. 2015; Rüdisser et al. 2015; Kuiper et al. 2013).

Obviously, the habitat suitability of different bird species to landscape structure varies depending on the group of species considered and the species assemblage for a given region (Atauri and de Lucio 2001). Some studies have shown varying responses of particular guilds to specific landscape metrics (Marja et al. 2013; Mimet et al. 2014; Rüdisser et al. 2015). With the results of our analysis, we introduce a methodology for (i) the differentiation of habitat requirements of guilds for typical habitats in rural areas based on landscape metrics and (ii) the identification of metrics characterizing the overlap in potential habitats of particular guilds using a holistic approach, as suggested, for example, by Marja et al. (2013).

Landscape metrics are widely accepted as a means to analyse the relationships between landscape structure and biodiversity (Schindler et al. 2013). The landscape metrics identified by our results as the most suitable for the differentiation of the preferred habitats between the chosen guilds are plausible in the context of the existing literature. In our results, the preferred areas for farmland birds were described best by Simpson’s diversity index, the shape index and the edge density set. The suitable areas can thus be described as basically consisting of only one habitat type, i.e. arable land, as not having highly complex shapes and as being rich in edges between the particular shapes. It is well known that skylarks, the most abundant farmland bird, avoid areas near forests (Fonderflick et al. 2013; Pätzold 1994) and landscapes with considerable forest cover or patches (Rüdisser et al. 2015). Field margins and transition zones were found to be the most attractive for skylarks (Kuiper et al. 2013) and corn buntings (Reino et al. 2009) as foraging and breeding areas. Josefsson et al. (2013) reported an apparent preference of skylarks for cropped areas adjacent to buffer strips. Grassy or herbaceous field margins are used preferentially by early breeding yellow wagtails, mainly for foraging (Gilroy et al. 2009).

The grassland species guild exhibited the lowest reclassification accuracy (66%) and the highest uncertainties of all guilds. This might be due to the lowest numbers of sightings of these bird species in our survey. The preferred areas for the grassland species were identified by the contiguity index distribution, patch density and shape index distribution set and are described as small, isolated grassland plots (low contiguity and low patch density) without highly complex shapes (low shape index). The occurrence of small closed drainage basins with grassland vegetation, which are extensively used or not managed at all, is a specific feature of many North-Middle European landscapes. The preference of the grassland birds for these features, as found in our study, might be strongly affected by the extensive management of such plots. The meadow pipit is known to inhabit open grassy areas with dense, low vegetation cover, such as extensive meadows, and to avoid very short grass in intensive meadows or grazed pastures (Kumstátová et al. 2004; Hötker 1989). The results of Vanhinsberg and Chamberlain (2001) suggest a mosaic of heather, bog and grassland as the optimum habitat for meadow pipits. The whinchat primarily inhabits cultivated grassland but with traditional extensive management (Fischer et al. 2013). In particular, early and frequent cutting is observed as the main driver for the recent population decline (Müller et al. 2005). The northern lapwing exhibits a preference for nesting in habitats with wet features (Eglington et al. 2010), which in our landscape were provided by the small drainage basins.

Habitat requirements for birds breeding in hedgerows could be distinguished with high statistical significance from the other bird guilds by applying the metric set of the edge density index, largest patch index and Simpson’s diversity index. The preferred habitats were the transient zones (edges) between the different habitat types (high Simpson’s diversity), in contrast to the forest habitats due to their small sizes (largest patches). Batáry et al. (2012) found clear differences in the bird community composition between hedgerow and forest edge habitats. The forest species were more abundant along the forest edges and only used hedges as a secondary or alternative habitat. The red-backed shrike was regarded as the most abundant hedgerow breeder in our study area, and Brambilla et al. (2009) found positive associations between shrike occurrences and both hedgerow length and partial shrub cover at the landscape and plot scale. Shrikes also showed a positive correlation with the presence of various habitat types in the vicinity of the hedges, such as grassland cover at the landscape level and herbaceous vegetation at the finer scale. The Eurasian chaffinch is partly classified as a forest species in other studies (Pelosi et al. 2014; Brambilla et al. 2015). Thus, most of the studies describe the Eurasian chaffinch’s preferred complex habitat structures as consisting of neighbouring forest (Pelosi et al. 2014), herbaceous ground and urban habitats (Brambilla et al. 2015) and especially the presence of fruit resources (Albrecht et al. 2012). Shake et al. (2012) found that many of the shrubland bird species in the United States were area-sensitive, while the patch shape index and the proportion of forest cover were insufficient predictors of occupancy by the shrubland birds.

Forest birds preferred areas with high shape index values, high Simpson’s diversity index values and low edge density values in our analysis. They can be differentiated from the hedgerow guild as preferring different habitat types, including forests (higher Simpson’s diversity index), that are of irregular shape (high shape index) and as avoiding transient zones (edges) between different habitat types. Within the agricultural matrix, forests are mostly singular patches and seldom share borders with other forest patches. In contrast, in forest-dominated landscapes, the forest patch area could be the most significant variable explaining the patch occupancy of residents and summering forest birds, as reported by Suk et al. (2014). Barbaro et al. (2014) reported that landscape diversity had a significant effect on forest bird functional evenness and dispersion, while native forest cover only affected evenness. However, the forest cover or distance to forest remnants is crucial for the conservation of particular guilds or functional groups in fragmented agro-forest landscapes (Tscharntke et al. 2008; Clough et al. 2009). There are strong structural differences between the forest interior and forest edges. Broughton et al. (2012) reported that the marsh tit occupation was lower within a 50-m edge buffer than within the forest interior. Edge avoidance is a typical response of specialist forest interior species (Burke and Nol 1998).

Bird species that prefer settlements and urban environments can be described with the same indices as the hedge breeding birds but with different factor levels. Settlement birds prefer the transient area between settlements and arable land (high edge density), the patchy environment around the settlements (high largest patch index LPI) and the high diversity between habitat types (high Simpson’s diversity index). Birds in urban environments can be classified into groups of distinct habitat requirements (Jokimäki and Suhonen 1998), especially regarding the density of buildings and trees or green space. The selected species in our case were considered as moderately urbanophilic species (Croci et al. 2008), and they require tree coverage (Redhead et al. 2013) and bare ground underneath. They also occur widely in a range of alternative habitats, including parkland, urban gardens and agricultural land (Hinsley et al. 2008). DeGraaf and Wentworth (1986) noted that suburbs vary in avian composition depending largely on the degree to which they alter natural habitats. Edge species can probably continue to thrive in suburbs. Sandström et al. (2006) found that the two tit species also used in our analysis were increasingly abundant towards the periphery of urban areas, while the sparrow was most abundant in highly diverse residential areas.

Our results demonstrate the sensitivity of the sets of landscape metrics to describe the potential occurrence of the particular bird guilds. In contrast to the results of Marja et al. (2013), who used three fixed landscape metrics at different scales, we identified sets of landscape metrics distinguishing all guilds with high classification accuracy (higher than 80%), except for the grassland species where the re-classification rate was only 66%. We cannot confirm the conclusion drawn by Uuemaa et al. (2013) in their review on the use of landscape metrics, i.e. that bird species generally respond more strongly to the composition of land use classes than to the configuration of landscapes. This statement seems to be related only to bird species diversity, not to habitat suitability for particular guilds. In our results, all groups of landscape metrics (composition, diversity, geometry and configuration indices) appeared in the final models for the particular guilds with changes in the variance explanation loadings. Simpson’s diversity index and the edge density served as explanatory variables for the habitat preferences of four of the five investigated guilds. Moreover, the role of particular landscape metrics seems to be quite sensitive to the selection of the target variable or to the definition of the guilds. Caprio et al. (2009) found that different response variables predicted the diversity of specialist or generalist forest birds. Edge density is regarded as a good predictor for bird abundances (Fletcher and Koford 2002; Rehm and Baldassarre 2007). Reino et al. (2009) drew attention to the fact that the edge response can vary between, for example, steppe and woodland bird guilds. The edge contrast even summarizes the effects of varying strengths, depending on the type of neighbouring habitats. Accordingly, Reino et al. (2009) distinguished between “hard” (habitat types with large qualitative distances) and “soft” (habitat types with low qualitative distances) edges. In our study area, the high number of arable field polygons determined the quality of the edge relationships. In many cases, the arable fields were adjacent to each other but were treated as individual habitats. This might explain the positive correlation of edge density with the farmland birds and the negative correlation with the forest and hedgerow guilds. The interpretation of edge density in isolation from the other metrics may lead to misinterpretations of the habitat models. Shake et al. (2012) reported that the area sensitivity was important for shrubland birds. Similar findings were reported for forest birds (Broughton et al. 2012; Burke and Nol 1998).

Many papers emphasize that the spatial scale affects the performance of the landscape metrics for terrestrial birds (Suk et al. 2014; Schindler et al. 2013; Oja et al. 2005; Morelli et al. 2013). Marja et al. (2013) showed that the variation explanation of landscape metrics for farmland bird variables increased with the size of the study area. Similarly, Böhning-Gaese (1997) revealed that the prediction of bird species richness changed with increasing raster sizes (grains). In particular, the habitat occupancy of habitat specialists and of rare species seems to improve at larger scales (Skórka et al. 2006). Morelli et al. (2013) demonstrated that scale effects varied indifferently for both predictor and response variables as well as for different landscape backgrounds. The authors suggested a radius between 125 and 250 m or between 100 and 5,000 m (Rüdisser et al. 2015) as the best trade-off between accuracy and variable bird home ranges for correlating landscape metrics with bird census data. The design of our bird survey and the calculation of the landscape metrics are in accordance with these recommendations. For conservation purposes, Morelli et al. (2013) suggested multiscale analyses, integrating the spatial distribution of niches and resources at smaller scales. According to our bird monitoring design, the spatial scale was pre-determined by the survey design having a fixed sample area for the field observations (250 m radius) and by the distance to the centre of the next sample area (500 m). To avoid inter-correlation of the data, analyses at larger scales are only possible by omitting data from intermediate overlapping sampling plots (in our case, every second plot). However, the model with reduced replications (*N*) would lose comparability with respect to the full models.

The lack of land use details might be the reason for the reduced model accuracy in our study with respect to the grassland birds. The evidence of this impact was clearly demonstrated for grassland birds in different European regions (Verhulst et al. 2004; Atkinson et al. 2005). Used or abandoned grasslands may have totally different habitat qualities for grassland birds. Morelli et al. (2013) suggested the use of multi-scale spatial models combining information on landscape structure with land use intensity at the plot scale to solve this problem. Similar findings have been reported for farmland (Wretenberg et al. 2010; Piha et al. 2007), forest (Villard et al. 1999) and hedgerow (Bonifacio et al. 2011) birds.

Our statistical analysis used LOP values for guilds within the sample areas for classification purposes. This brings some advances to the commonly used presence/absence information of representatives of the guilds (Mimet et al. 2014; Morelli et al. 2013; Oja et al. 2005). We did not use the number of observed individuals in the sample areas in order to avoid influences originating from species-specific home ranges, breeding densities and social behaviour (e.g. flocking), and extreme observation values. Therefore, it is possible that different observation situations lead to identical LOP values. This is not considered as a serious restriction because the distinction between preferred and less preferred habitats should be based on the persistence of the incidence of guild representatives rather than the number of individuals.

To derive qualitative relationships between the occurrence of breeding bird guilds and information concerning the landscape structure, binary logistic regression was applied as a binary classifier. This method is well adopted in habitat selection studies (Keating and Cherry 2004; Jokimäki et al. 2014). One striking advantage of this method in landscape-related research is the option to combine inputs of different scale levels without further requirements concerning their statistical properties. It is true that the binary logistic regression in the applied form merely generates dichotomous class memberships, but it is possible to evaluate the relative importance of the selected input landscape metrics and their statistical significance. Furthermore, it is possible to refine the current approach with additional input variables (e.g. crop diversity, site conditions). If a finer classification is needed, input and target data could be reclassified to apply a multinomial logistic regression.

It cannot be expected that the detailed results of this study are transferable to other regions without modifications. The derived results are, to a certain degree, specific to the Quillow investigation area. This holds for the definition of the guilds and for the derived dependencies between bird occurrence and landscape structure. Therefore, when moving to other regions, it will certainly be necessary to redefine the guild composition depending on local abiotic situations and land use, that is, the general approach to assess habitat suitability can be retained, but it will require local adaptation.

Finally, the mutual correlations and context sensitivity of single landscape metrics call for multifaceted (Rüdisser et al. 2015) and holistic methodological approaches that can be adapted to specific landscape conditions (Caprio et al. 2009). We suggest the consideration of a wide range of species classified into guilds to represent the bird community of a landscape and different types of landscape metrics. There is an important link that needs to be further explored between the metrics of bird functional diversity and the ecosystem services provided by avian predation (Uuemaa et al. 2013; Gonzalez-Gomez et al. 2006; Philpott et al. 2009).

## Conclusions

In our paper, we introduce an operational approach to assess the habitat suitability of agricultural landscapes for farmland birds on the basis of landscape metrics. Our aim is to develop an assessment method applicable to a wide range of both landscapes and bird communities. Therefore, we build on a comprehensive consideration of both the whole bird community and the whole landscape through combining the procedures of “guild classification” for the bird species and of “landscape metrics” for the analysis of the structural characteristics of landscapes. The applicability of the novel assessment method was verified by empirical bird monitoring data for a landscape in north-east Germany carried out over a 4-year period with more than 20,000 individual bird observations. The resulting algorithms allow for the discrimination of the habitat requirements for four of the analysed five bird guilds. Finally, a subset of six landscape metrics out of the overall tested 16 metrics variables proved to be meaningful and sufficient for creating habitat suitability maps for the whole landscape and all bird guilds considered. The potential advantages of this approach include its applicability to (i) landscapes with different natural pre-conditioning (setting) and to (ii) different bird fauna compositions (inventories), which we suggest to investigate further.

## Electronic supplementary material

ESM 1 (PDF 7018 kb)
