# Supplementary material for: Assessing the habitat suitability of agricultural landscapes for characteristic breeding bird guilds using landscape metrics
Source: Environ Monit Assess. 2017 Mar 16;189(4):166. doi: 10.1007/s10661-017-5837-2 (PMC5355513; doi:10.1007/s10661-017-5837-2)
Supplement: Supplementary file 1 — (PDF 7018 kb) [file 10661_2017_5837_MOESM1_ESM.pdf]

Environmental Monitoring and Assessment

Assessing the habitat suitability of agricultural landscapes for characteristic breeding bird guilds using landscape metrics

Electronic Supplementary Material

Friederike Borges[1,2]*, Michael Glemnitz[1], Alfred Schultz[2], and Ulrich Stachow[1]

[1]Leibniz Centre for Agricultural Landscape Research (ZALF), Eberswalder Str. 84, 15374 Müncheberg, Germany

[2]Eberswalde University for Sustainable Development, Alfred-Möller Str. 1, 16225 Eberswalde, Germany

*Email: Friederike@Borges.de

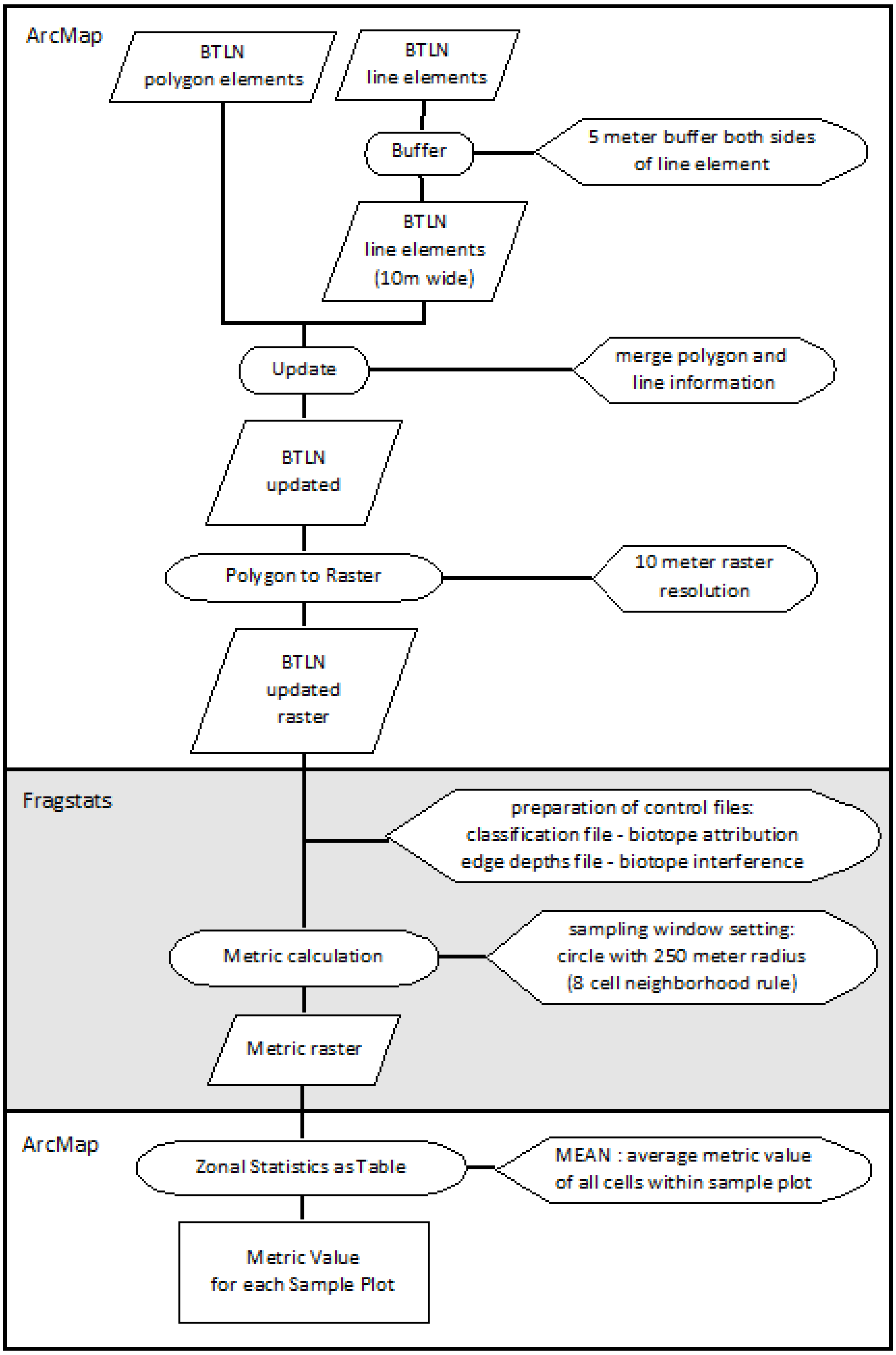

Fig. A 1 Workflow of the spatial data preparation and pre-processing to compute landscape metric calculations and generate index values for the entire observation area; calculating metric values for each sample area for the analytical statistics with the ArcGIS tool *Zonal Statistics as Table* (BTLN: Comprehensive Biotope- and Land use Map of the Federal State of Brandenburg)

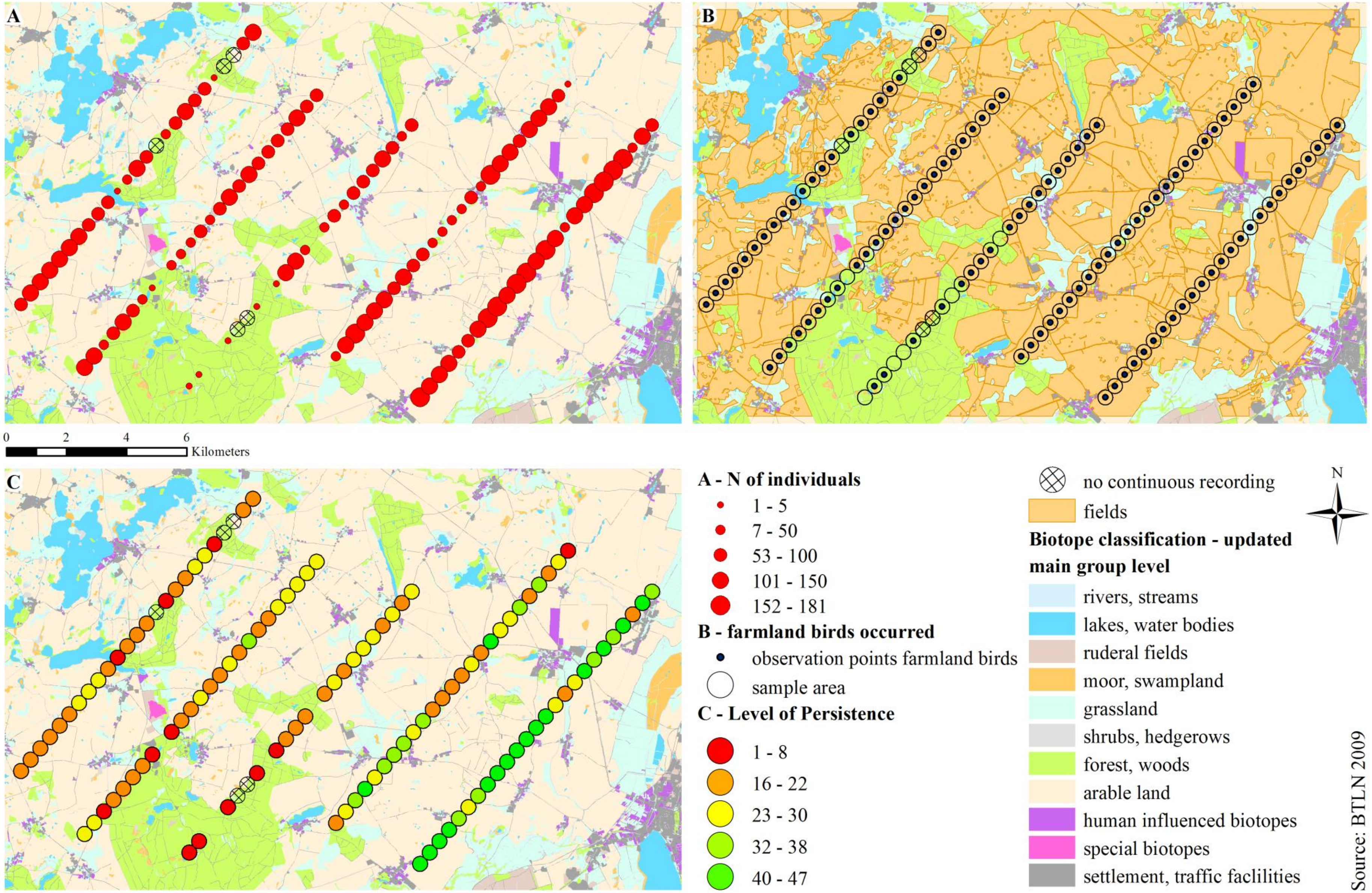

Fig. A 2 Distribution maps of farmland guild occurrence: A: N of individuals observed at each sample area over entire monitoring period; B: sample areas where at least one of the guild species were detected plotted against the assumable preferred habitat (arable land); C: Level of Persistence (LOP) of the farmland guild (LOP = N of species of guild * N of surveys species observed -> LOP^{max}= 3 * 19 = 57)

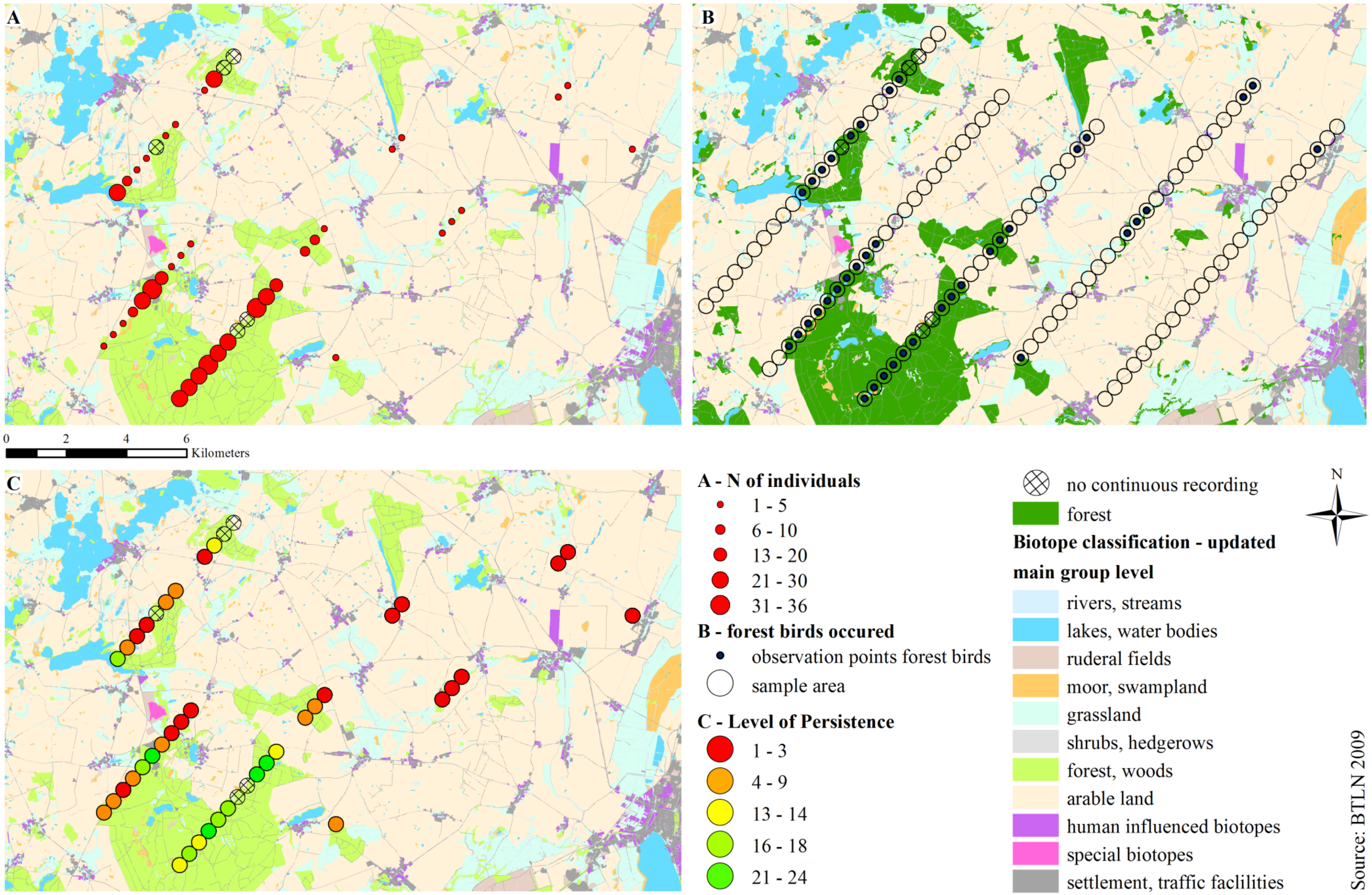

Fig. A 3 Distribution maps of forest guild occurrence: A: N of individuals observed at each sample area over entire monitoring period; B: sample areas where at least one of the guild species were detected plotted against the assumable preferred habitat (forest); C: Level of Persistence (LOP) of the forest guild
(LOP = N of species of guild * N of surveys species observed -> LOP^{max}= 3 * 19 = 57)

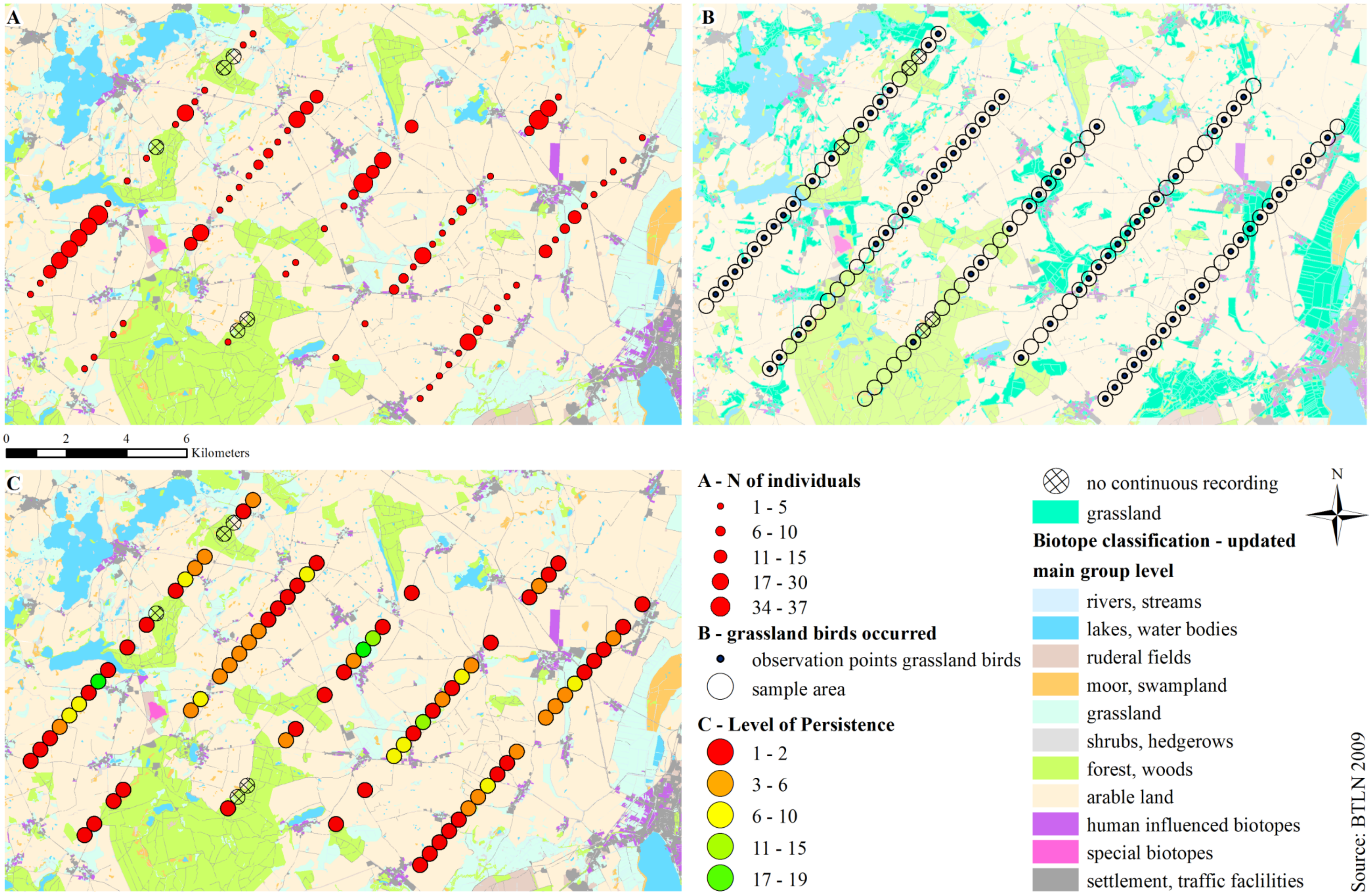

Fig. A 4 Distribution maps of grassland guild occurrence: A: N of individuals observed at each sample area over entire monitoring period; B: sample areas where at least one of the guild species were detected plotted against the assumable preferred habitat (grassland); C: Level of Persistence (LOP) of the grassland guild (LOP = N of species of guild * N of surveys species observed -> LOP^{max}= 3 * 19 = 57)

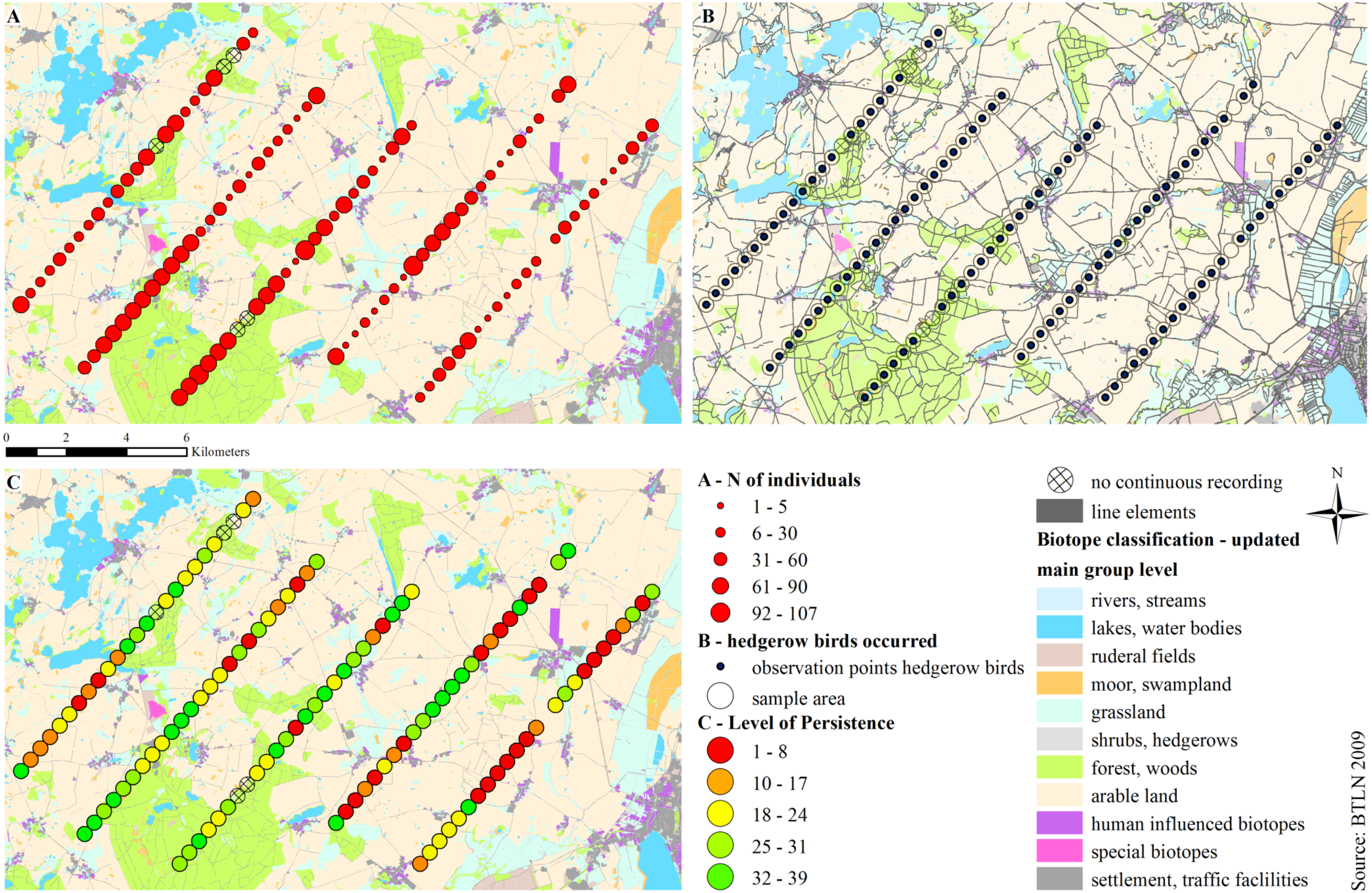

Fig. A 5 Distribution maps of hedgerow guild occurrence: A: N of individuals observed at each sample area over entire monitoring period; B: sample areas where at least one of the guild species were detected plotted against the assumable preferred habitat (shrubs and hedgerows); C: Level of Persistence (LOP) of the hedgerow guild (LOP = N of species of guild * N of surveys species observed -> LOP^{max}= 3 * 19 = 57)

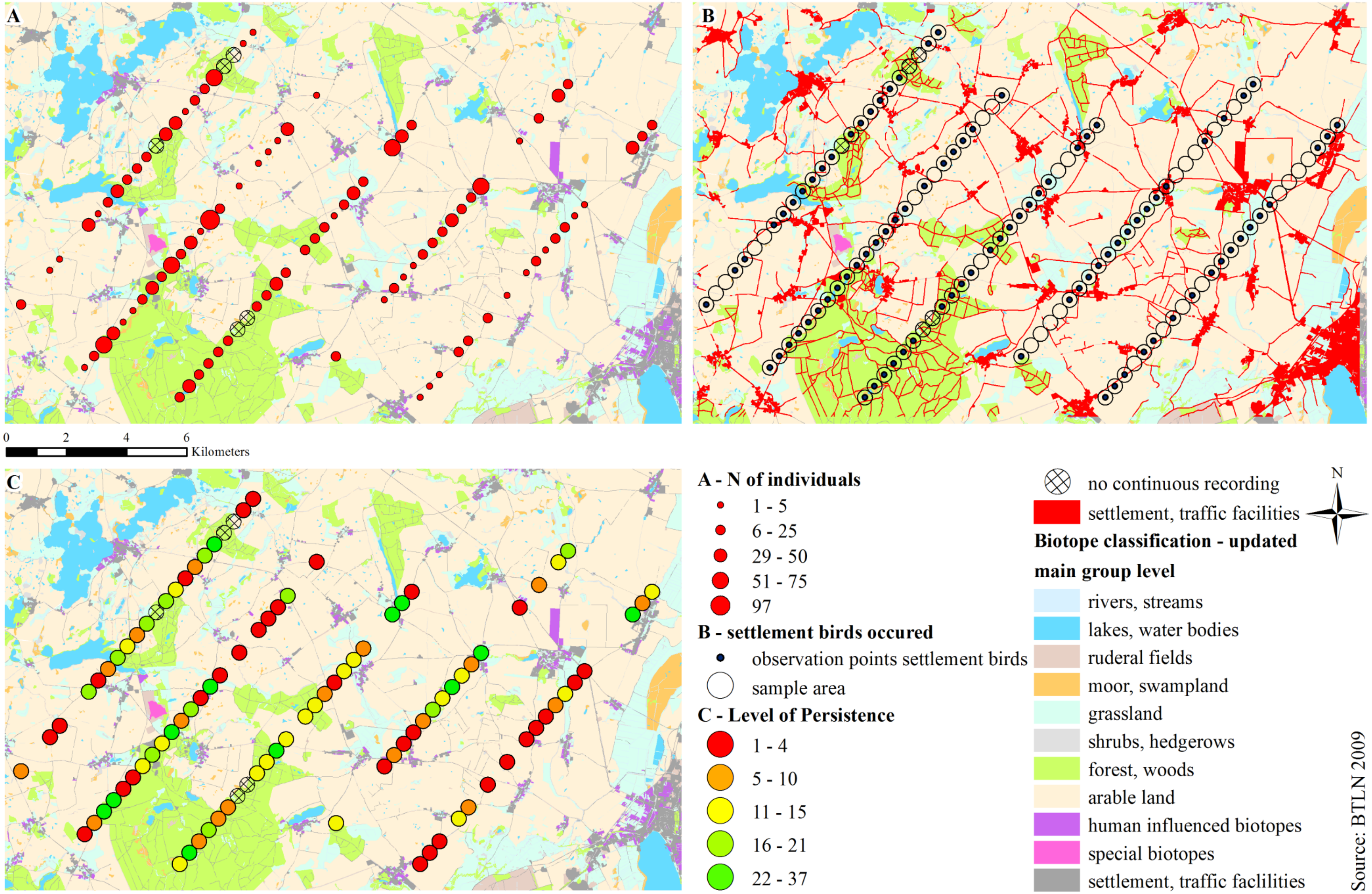

Fig. A 6 Distribution maps of settlement guild occurrence: A: N of individuals observed at each sample area over entire monitoring period; B: sample areas where at least one of the guild species were detected plotted against the assumable preferred habitat (settlement); C: Level of Persistence (LOP) of the settlement guild (LOP = N of species of guild * N of surveys species observed -> LOP^{max}= 3 * 19 = 57)

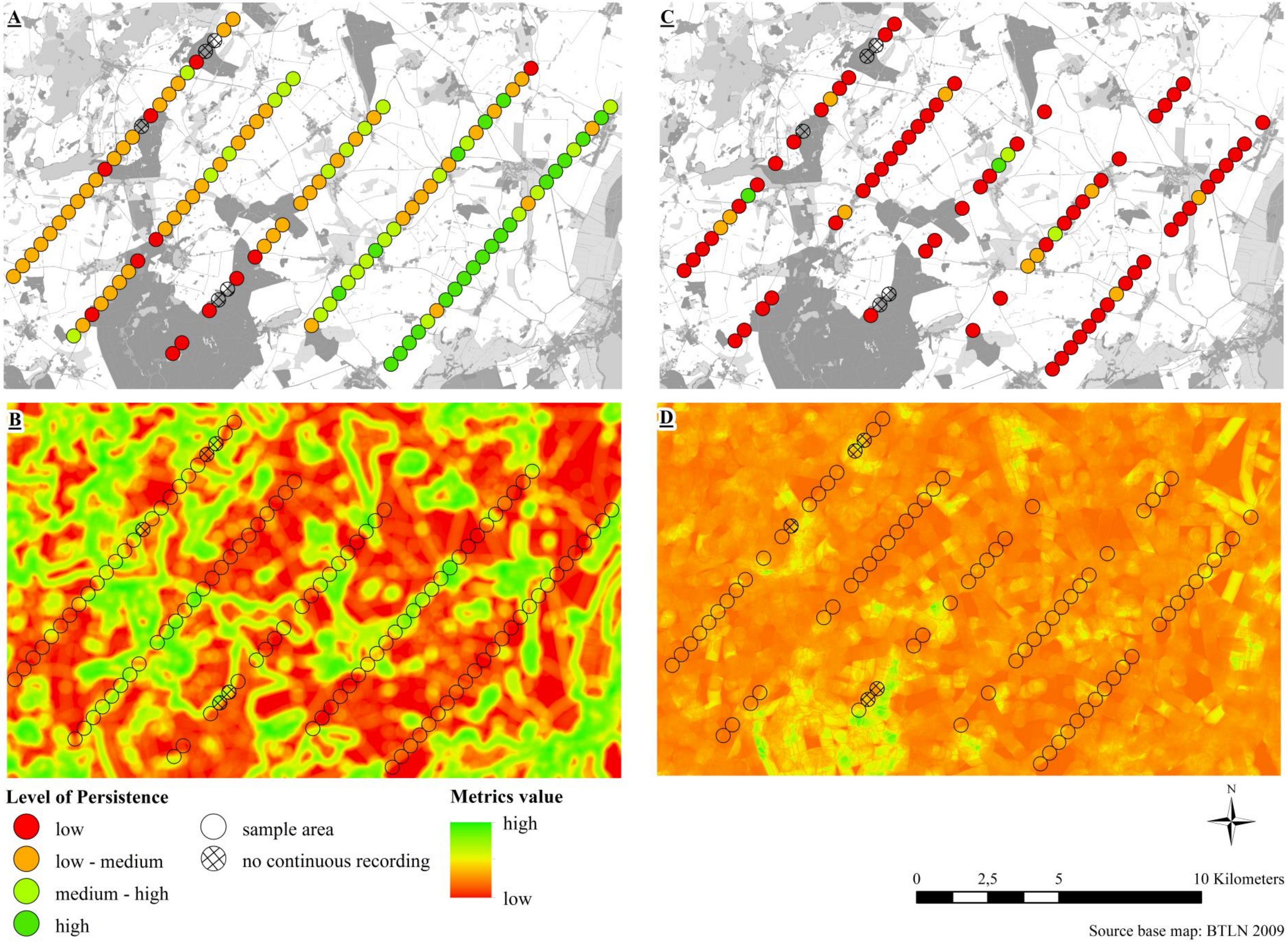

Fig. A 7 Distribution maps for the farmland (A, B) and grassland guilds (C, D) occurrence in the observation area in combination with landscape metric maps: A: Level of Persistence (LOP) for the farmland guild; B: LOP of farmland birds occurrence plotted against the Simpson´s diversity index landscape metric; C: LOP for the grassland guild; D: grassland birds occurrence plotted against the shape mean landscape metric. (Sample areas with LOP=0 are not visualised)
